# The Story of the Insane from Year to Year

**Published:** 1894-02-10

**Authors:** 


### THE STORY OF THE INSANE FROM YEAR TO YEAR.

(Sixth Series.)

### LANCASHIRE COUNTY ASYLUM, PRESTWICH.

This, the largest asylum in England, had under treatment on the last day of the year the enormous number of 2,335 patients. The admissions were 583, the recoveries 309, and the deaths 183. Thrown into percentages in the usual way, we find the recoveries come out 50 per cent. and the deaths at 5·93 per cent. The formei is much above the average and the latter much below it. Intemperance in drink caused the insanity in 95 of the year's admissions, and hereditary influence accounted for 126. Of the deaths, 83 were due to brain mischief, in one form or other, and 38 to consumption. The asylum is now full, and a large number of patients have been refused admission. Although Lancashire is in possession of four gigantic county asylums, this pressure is in every division of the county, and it is proposed to build another for 2,000 beds, and a smaller one is about to be commenced for the borough of Blackburn. It would seem that the average annual increase for the whole county is, for the last ten years, 243. It will take four years to build the new asylum. There will, therefore, be 1,000 additional lunatics to provide for, and these, with the numbers at present oscillating between asylum and workhouse, will be sufficient to fill the new building on the day of its opening. Attendants Eastwood and Hamilton have retired on their pensions, and deserve our congratulations. There are now eight assistant medical officers. The weekly cost of maintenance was 9s. per head.

### JOINT COUNTIES ASYLUM, CARMARTHEN.

There were resident on the last day of the year 556 patients, being an increase of four only during the year. There were 105 admissions, 34 recoveries, and 55 deaths. As usual the chief canses of insanity were drink and hereditary influence; the former accounting for 17 of the admissions, and the latter for 27. "Previous attacks" account for 23. Nineteen of the deaths were due to brain diseases and 8 to consumption. The recoveries stand at 33·3 per cent. and the deaths at 9·82. It is not often that medical superintendents have to chronicle deaths among the attendants, but two of Dr. Hearder's staff died during the year. They had been incapacitated from duty for some time, and we are much pleased to learn that the committee allowed them to remain to be nursed in the asylum. Dr. Hearder also mentions that an attendant was injured by a patient. If an attendant in a moment of impulse after being ill-treated should strike a patient everyone hears of it, but when the opposite happens it is borne in silence, although the latter assaults are probably a hundred times as numerous as the former. The visitors say they "have pleasure in again bearing testimony to the efficient management of the institution under Dr. Hearder." The Commissioners say there is evidence of overcrowding, but the visitors in their report say nothing of the accommodation, although, if we remember rightly, this is one of the matters which their report should contain. The weekly cost per head is 7s. 11¼d.

### NORFOLK COUNTY ASYLUM.

On December 31st, 1892, there were in this asylum 768 patients, being an increase of 28 during the year. The admissions were 178, the recoveries 69, and the deaths 70. These numbers yield the following percentages: Recoveries 42·33, deaths 9·34. Hereditary influence caused the insanity in 62 of the admissions; previous attacks caused it in 48, and drink in 13. Table IV. shows that 31 of the deaths were due to brain diseases, 1 to typhoid fever, and 12 to consumption. Dr. Thomson's report is very clear and well arranged; and the asylum would seem to be marching forward in many directions. Dysenteric diarrhœa, which was at one time endemic, is now very rare, being limited during the year to three cases. There were three cases of typhoid fever, however, and one of those died. Dr. Thomson says he "is unable to assign any cause in these cases. The water is pure, and the drains sound, and the occurrence of these cases is very puzzling." Other medical superintendents have been equally puzzled to account for sporadic cases of typhoid and dysentery; and there would seem to be a disease clinically identical with enteric fever, but showing no tendency to spread. Indeed we believe this disease has been mentioned by one author at least. The Committee say they have much satisfaction in "again according their satisfaction with the efficient manner in which Dr. Thomson has managed the establishment." The weekly cost per head is 9s. 4d.

### GRAHAMSTOWN ASYLUM, CAPE OF GOOD HOPE.

There were 186 patients in residence on January 1st, 1892, and by the end of the year the numbers had risen to 208. The admissions reached 130, the deaths 27, and the recoveries 47. The recoveries work out at 36·15, and the deaths at 13·83. Both of these are higher than their own average. Drink was the cause of the insanity in 12 cases, and hereditary influence in 23. There is a curious mixture of nationalities in the population of the asylum. We find English, Scotch, Dutch, Irish, Germans, Swedes, Russians, Hottentots, Fingoes, and Kaffirs. It would be interesting to know how these various races and nationalities get on together. What would an opponent of sedatives say to this: "The natives are very fond of dancing and singing, particularly during nights when the moon is full; this, which is not an insane sympton at all, although to us it may seem unnatural, frequently prevents my European patients obtaining their night's rest, and I am therefore compelled to administer sedative draughts to the noisy natives." For ourselves, we should probably do as Dr. Greenlees does. Many improvements have been carried out, and many others are contemplated. Dr. Greenlees thinks that more single-bedded rooms are required; indeed, he is of opinion that each native should have a room to himself. The maintenance rate has been reduced from 2s. 2½d. per diem to 1s. 11½d.

### ARGYLL AND BUTE ASYLUM.

This district asylum contained 373 patients at the close of the year 1892, being an increase of two only during the year. There were 69 admissions (including 13 re-admissions), 31 recoveries, and 22 deaths. The recoveries come out about the average of public asylums, being 39 per cent. on the admissions, while the death-rate is extremely low, being only 4 per cent. on the average number resident. Hereditary influence caused the insanity in 14 cases, and drink in 4. Five of the deaths were caused by brain diseases, and 6 by consumption, while pneumonia and congestion of the lungs account for another 6. Scarlet fever broke out twice among the families living on the estate, and in neither case could the source of infection be traced. The Committee say, "The asylum continues to be conducted in a satisfactory manner, under the skilful superintendence of Dr. Cameron," and the Commissioners say that all parts of the house were found in excellent order. The weekly cost of maintenance is 8s. 8½d.

